# Analyzing Kinase Similarity in Small Molecule and Protein Structural Space to Explore the Limits of Multi-Target Screening

**DOI:** 10.3390/molecules26030629

**Published:** 2021-01-26

**Authors:** Denis Schmidt, Magdalena M. Scharf, Dominique Sydow, Eva Aßmann, Maria Martí-Solano, Marina Keul, Andrea Volkamer, Peter Kolb

**Affiliations:** 1 Institut für Pharmazeutische und Medizinische Chemie, Heinrich-Heine-Universität Düsseldorf, 40225 Düsseldorf, Germany; denis.schmidt@uni-duesseldorf.de; 2 Pharmaceutical Chemistry, Philipps-University Marburg, Marbacher Weg 6, 35037 Marburg, Germany; magdalena.scharf@pharmazie.uni-marburg.de (M.M.S.); mariamartisolano@gmail.com (M.M.-S.); 3 In Silico Toxicology and Structural Bioinformatics, Institute of Physiology, Charité—Universitätsmedizin Berlin, Charitéplatz 1, 10117 Berlin, Germany; dominique.sydow@charite.de (D.S.); eva.assmann@mail.de (E.A.); 4 Chemical Biology, Technical University Dortmund, Otto-Hahn-Str. 4a, 44227 Dortmund, Germany; marina.keul@tu-dortmund.de

**Keywords:** multi-target ligands, docking, chemoinformatics, bioinformatics, kinases, binding site comparison, anti-target

## Abstract

While selective inhibition is one of the key assets for a small molecule drug, many diseases can only be tackled by simultaneous inhibition of several proteins. An example where achieving selectivity is especially challenging are ligands targeting human kinases. This difficulty arises from the high structural conservation of the kinase ATP binding sites, the area targeted by most inhibitors. We investigated the possibility to identify novel small molecule ligands with pre-defined binding profiles for a series of kinase targets and anti-targets by in silico docking. The candidate ligands originating from these calculations were assayed to determine their experimental binding profiles. Compared to previous studies, the acquired hit rates were low in this specific setup, which aimed at not only selecting multi-target kinase ligands, but also designing out binding to anti-targets. Specifically, only a single profiled substance could be verified as a sub-micromolar, dual-specific EGFR/ErbB2 ligand that indeed avoided its selected anti-target BRAF. We subsequently re-analyzed our target choice and in silico strategy based on these findings, with a particular emphasis on the hit rates that can be expected from a given target combination. To that end, we supplemented the structure-based docking calculations with bioinformatic considerations of binding pocket sequence and structure similarity as well as ligand-centric comparisons of kinases. Taken together, our results provide a multi-faceted picture of how pocket space can determine the success of docking in multi-target drug discovery efforts.

## 1. Introduction

Small-molecule modulators of protein function are the most frequent type of molecules in use for the treatment of diseases due to their favorable pharmacokinetic properties [1]. Such ligands bind to cavities on protein surfaces—the binding sites—and compete with substrates or native ligands, or they alter the protein conformation. For such a molecule to become an efficacious drug, it has to possess adequate affinity for its protein target, solubility, membrane permeability and stability. Furthermore, its overall binding profile has to be compatible with its intended mode of action. On the one hand, unintended binding to proteins other than the primary target can cause side effects. On the other hand, several diseases require the simultaneous modulation of multiple proteins in order to be treated successfully [2,3,4]. While the binding profile of a ligand can certainly be engineered through medicinal chemistry, starting out from a scaffold or a molecule that already displays the desired affinities towards several target proteins can only be advantageous. Successful approaches to identify dual-selective compounds by means of docking have been published before [5,6,7]. In this work, we were interested to see whether this docking-based approach can be broadened beyond what has previously been done by applying it to more than two proteins and by also including anti-targets.

Our target family of choice for this study were kinases, which are established drug targets to combat cancer and inflammatory diseases [8]. They play a major role in signal transduction by phosphorylating other proteins and are frequently mutated in tumors [9,10]. The human kinome consists of over 540 protein kinases that were clustered by Manning et al. [11] into eight major groups, e.g., tyrosine kinases (TKs), based on overall sequence similarity. The interest in this protein family has resulted in the generation of a wealth of freely available compound, bioactivity and structural data, which can be used for computer-aided analysis and guidance in drug design [12]. Such data have also successfully been applied to develop predictive models [13]. As of July 2020, there are 4864 X-ray structures of human kinases available in the PDB [14] (number obtained from KLIFS, an open-source database for kinase–ligand interaction fingerprints and structures, [15,16,17]) and 53 small molecule kinase drugs (only counting ‘-nibs’) have made it to FDA approval [18]. Most of the approved drugs bind to the ATP-binding pocket and its immediate surroundings, which include important regions like the hinge region (forming key hydrogen bonds), the DFG motif, the αC-helix and the glycine-rich (G-rich) loop. They either block the active state of the kinase or lock the protein in an inactive state. In the active state, the DFG motif’s phenylalanine (F) is pointing into the hydrophobic pocket, while the aspartate (D) coordinates a magnesium ion for ATP binding (DFG-in conformation). Additionally, conserved residues in the αC-helix and β3-sheet form a salt bridge (αC-in conformation) and the G-rich loop stabilizes ATP. Different descriptors have been defined to classify activity states based on these structural properties [15,16,19,20]. Since kinases share a similar fold—especially in the active site—most kinase inhibitors suffer from promiscuous binding. Sunitinib, for example, was found to bind to more than 50% of a panel of 290 kinases [21]. Similarly, dasatinib binds to a broad spectrum of TKs with high affinity [21]. Such promiscuous binding has been related to several of the side effects of current kinase inhibitors. Taken together, these facts clearly demonstrate the need for methods which are able to filter out compounds binding to kinases considered as anti-targets in order to facilitate the design of more selective kinase inhibitors.

In this study, we investigated the possibility to design and identify ligands with a defined polypharmacology through structure-based approaches. To that end, we docked identical molecule sets against multiple kinase targets to identify novel kinase inhibitors with defined rationally-selected profiles. Importantly, the resulting hits were not only selected for their ability to simultaneously bind to multiple kinase targets but also specifically filtered to avoid an established kinase anti-target. We also used available kinase-focused data to analyze different facets of kinase similarity in an attempt to evaluate the likelihood with which certain kinase combinations can be targeted simultaneously or individually. We evaluated the similarity of the binding sites based on the correlation of the docking ranks of the individual molecules, i.e., we considered binding sites to be similar when they were predicted to bind the same compounds in a similar docking rank order. Moreover, we assessed the congruence of this ranking with other ligand-centric as well as protein sequence- and structure-based measures. We evaluated our docking calculations by predicting selective as well as multi-target ligands (with defined targets and anti-targets) for three triplets of kinases and tested these predicted ligands experimentally. In this way, we identified and validated a sub-micromolar dual inhibitor of EGFR and ErbB2, with no activity against BRAF as the anti-target.

The results of our study allow us to reflect on the similarity boundaries determining the suitability of structure-based drug design (SBDD) to successfully address a specific multi-target combination. In particular, they show the necessity for ever-larger libraries that hold diverse molecules, in order to increase the likelihood of identifying ligands tailored towards predefined selectivity profiles.

## 2. Results and Discussion

Herein, the selected kinase profiles are rationalized first and the virtual screening results against these panels are discussed. Then, the experimental results for the selected compounds are presented. Finally, the similarity between the kinases of the studied profiles is analyzed with respect to different ligand- and protein-centric measures.

### 2.1. Kinase Profiles

We focused our analysis on a target panel comprising kinases with medical relevance as well as a typical anti-target, known to be associated with frequent side effects of kinase inhibitors. All kinases in this set have been thoroughly characterized in the literature and are summarized in Table 1.

The Erythroblastic leukemia viral oncogene homolog (ErbB) subclass of Receptor Tyrosine Kinases (RTKs) consists of four members named from ErbB1 (better known as epidermal growth factor receptor [EGFR]) to ErbB4 and they bind the EGF family of peptides with their extracellular region [22]. The ErbB family is involved in the regulation of a multitude of signaling pathways associated with cell development. It is thus not surprising that aberrant ErbB signaling occurs in many cancers. Of note, patients with altered EGFR and ErbB2 expression suffer from a more aggressive disease. Especially breast cancer overexpressing ErbB2 is associated with poor patient prognosis [23]. Unfortunately, therapy is often effective only for a short time and tumors will escape inhibition by activating pathways downstream of ErbB receptors via other kinases. This has been demonstrated for the phosphatidylinositol-3-kinase (PI3K) pathway, which is directly or indirectly activated by most ErbBs [24]. After initial downregulation of PI3K activity upon inhibition of ErbBs, this pathway often recovers. Combination therapies are used to circumvent this problem, albeit with limited success. There is also evidence that tumor cells escape the negative effects of EGFR inhibition by upregulating tumor angiogenesis-promoting growth factors. A study used two antibodies against EGFR and VEGFR2 (vascular endothelial growth factor receptor 2), respectively, to treat gastric cancer grown in nude mice [25]. The combination resulted in significantly greater inhibition of tumor growth.

Based on these experimental observations, we aggregated the investigated kinases in “profiles” (Table 2). Profile 1 combined EGFR and ErbB2 as targets (indicated by a ‘+’) and BRAF (from rapidly accelerated fibrosarcoma isoform B) as a (general) anti-target (designated by a ‘—’). Out of similar considerations, Profile 2 consisted of EGFR and PI3K as targets and BRAF as anti-target. This profile is expected to be more challenging as PI3K is an atypical kinase and thus less similar to EGFR than for example ErbB2 used in Profile 1. Profile 3, comprised of EGFR and VEGFR2 as targets and BRAF as anti-target, was contrasted with the hit rate that we found with a standard docking against the single target VEGFR2 (Profile 4).

To broaden the comparison and obtain an estimate for the promiscuity of each compound, the kinases CDK2 (cyclic-dependent kinase 2), LCK (lymphocyte-specific protein tyrosine kinase), MET (mesenchymal-epithelial transition factor) and p38α (p38 mitogen activated protein kinase α) were included in the experimental assay panel and the structure-based bioinformatics comparison as commonly used anti-targets.

### 2.2. Virtual Screening against Kinase Profiles

Following our previous approach to identify ligands with tailored selectivity profiles by virtual screening [6], the aim of this study was to evaluate the possibility to add anti-targets to a kinase profile. We hence modified our previous approach to incorporate profiles with more than two kinases, multiple structures per kinase and the selection of targets and anti-targets (Equation (1) in Section “Data and Methods”).

Starting from the EGFR/ErbB2 pair, we included BRAF as a promiscuous anti-target, resulting in Profile 1 (see Section 2.4.1 for a discussion of promiscuity values). We therefore prioritized molecules with high rank (i.e., favorable docking scores) in EGFR and ErbB2 as well as low rank (i.e., unfavorable docking interactions) in BRAF. The ZINC lead-like and ZINC drug-like subsets, containing 4.6 and 10.6 million molecules, respectively, were docked into each of the selected structures of these kinases (cf. “Data and Methods”). After docking the smaller lead-like subset to EGFR, ErbB2 and BRAF, the kinases comprising Profile 1, we identified a high mutual overlap in terms of well-ranked compounds between these three kinases (6982 common compounds in the top-ranked 25,000 compounds for EGFR and ErbB2, 4732 for ErbB2/BRAF and 4675 for EGFR/BRAF, respectively, each number representing the maximum over all pairwise comparisons of all docking runs of the lead-like ZINC subset into the different structures of these kinases). Thus, many promising poses in EGFR/ErbB2 were invalidated by a high-rank in the anti-target BRAF. Therefore, we deemed the docking of the larger drug-like subset necessary to obtain a sufficient number of poses with reasonable binding modes to select from after re-ranking. The re-ranking procedure was devised to prioritize molecules matching the requested profile, i.e., molecules with favorable docking rank in all targets but unfavorable docking ranks in all anti-target structures (see “Data and Methods” for details). Finally, we selected 18 molecules (see Table 2 and Table S1) based on visual inspection for this profile (see “Data and Methods” for more detail) from the re-ranked lists of both molecule sets and evaluated these experimentally.

Similarly, for Profile 2, using EGFR and PI3K as targets and again BRAF as an anti-target (Table 2), we docked both the ZINC lead-like as well as the drug-like subsets. Again, we deemed the drug-like subset to be necessary due to the large overlap of the top-scoring lead-like molecules of the targets with the ones ranked favorably in the anti-target (4683, 4675 and 6591 for EGFR/PI3K, EGFR/BRAF, and PI3K/BRAF, respectively). For this profile, we selected nine molecules (Table 2 and Table S1).

The parallel docking calculations for Profiles 3 and 4 (Table 2) yielded eight and four candidate ligands, respectively (Table 2 and Table S1). For Profile 3, the number of common molecules in the top 25,000 was 4610 and 5544 for VEGFR2/EGFR and VEGFR2/BRAF, respectively. As above, the overlap between EGFR and BRAF was 4675.

### 2.3. Experimental Validation

In total, 24 compounds selected from Profiles 1 and 2 (Table 2 and Table S1) were tested in the DiscoverX assay against kinases EGFR, ErbB2, BRAF, VEGFR2, LCK, CDK2, MET, p38α and PI3K (Table S2), as well as in an additional confirmatory assay by Eurofins against EGFR, ErbB2, BRAF and PI3K (Table S3). Only one of the 24 compounds, DS39984, showed measurable binding to the desired kinases (Profile 1, Table 3 and Tables S1–S3), while binding to neither Profile 1’s anti-target BRAF nor any of the other tested kinases (VEGFR2, CDK2, LCK, MET, p38α and PI3K). This compound DS39984 emerged from the screening campaign against Profile 1 (+EGFR+ErbB2—BRAF) and was picked from the drug-like subset of the ZINC database. We further validated the binding of this ligand and determined binding curves in an independent assay with IC${}_{50}$ values of 324 and 220 nM (note that both enantiomers were docked—with the R-enantiomer more favorably ranked, but the racemate was tested) against EGFR and ErbB2insYVMA (a variant of ErbB2 with an insertion of four residues distant from the binding pocket), respectively (Table 3, Rauh Lab).

**Figure 1:**
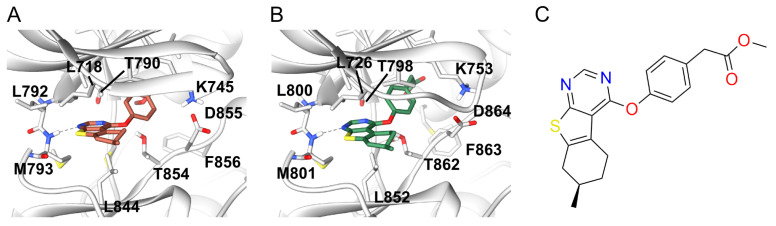
Docking poses of the R-enantiomer of compound DS39984 bound to (**A**) EGFR (PDB 3POZ, DFG-in) and (**B**) ErbB2 (PDB 3PP0, DFG-in); and (**C**) 2D representation of DS39984. The protein structure is shown as cartoon, colored in grey, the compound as sticks. Interacting binding site residues are represented as sticks and labeled. Hydrogen bonds between protein and ligand are indicated by dark gray dashed lines.

As shown in the predicted binding modes in EGFR and ErbB2 (Figure 1), DS39984 adopts a similar binding orientation in both proteins, with the pyrimidine portion forming a hydrogen bond to the hinge region. The methylester moiety is oriented more towards the back of the binding pocket, where both kinases feature rather voluminous cavities. This predicted binding mode to the hinge region is consistent with the sensitivity of DS39984 towards the T790M mutation: Affinity for the EGFRL858R/T790M double mutant is abolished (IC${}_{50}>10$ µM), whereas the affinity for the EGFRL858R mutant is 2351±397 nM. In contrast, in both BRAF structures used herein, the predicted poses are flipped and have their methylester moiety pointing towards the solvent (Figure S1). A similar hinge binding interaction as in EGFR and ErbB2 is only present in one of the two poses (in the docking to BRAF structure 1UWH). This occurs despite the fact that in the 1UWH crystal structure the deep back pocket is open due to the crystallized ligand. Thus, in principle, a binding mode of DS39984 similar to the ones predicted in EGFR and ErbB2 is not per se excluded in BRAF due to steric reasons.

Note that DS39984 is not present in ChEMBL and has low similarity to known kinase ligands in ChEMBL (no ligand with Tanimoto similarity >0.7 as implemented in the ChEMBL web interface as of 18 October 2020). Furthermore, none of the additionally tested kinases (LCK, CDK2, MET and p38α) were inhibited by the molecule, which underlines, together with absence of BRAF inhibition, the potential of DS39984 as a novel, selective nanomolar EGFR and ErbB2 inhibitor.

Eight compounds were selected for Profile 3 (+EGFR+VEGFR2—BRAF, Table 2 and Table S1) and tested in the DiscoverX assay against EGFR, VEGFR2, BRAF and ErbB2. However, none of the compounds exhibited a relevant effect against any of these kinases. To crudely estimate the ligandability of VEGFR2, we docked against this target individually (Profile 4). However, we did not observe many poses that passed our visual inspection (see “Data and Methods” for details) and were able to select only four compounds from the docking to VEGFR2. These were tested in the same assay. Again, none of these compounds showed an effect on VEGFR2 activity. While the number of tested compounds is certainly too small to draw clear conclusions, the fact that only few compounds could be considered in the first place and that those few were inactive might indicate that VEGFR2 is more challenging with respect to the identification of ligands by docking than for example EGFR and ErbB2. One explanation for this could be associated with the fact that the vast majority of VEGFR2 structures show DFG-out(like) conformations (ratio DFG-in/out(like) structures in the PDB: 5/34 for VEGFR2 compared to 168/22 for EGFR, as of KLIFS 25 November 2020). Note that several FDA-approved kinase inhibitors bind to DFG-out(like) VEGFR2 conformations, e.g., axitinib, sunitinib and sorafenib [26]. In contrast, we used DFG-in conformations of VEGFR2 for docking in order to maximize comparability with the other kinase structures used.

Unexpectedly, however, we found that one of these four compounds selected for VEGFR2 inhibition, K001MM011, actually inhibited EGFR and, to a lesser extent, ErbB2 (Table 3 and Table S2). While K001MM011 was picked from the docking to VEGFR2 only, we retrospectively inspected the ranking of this compound in the docking to EGFR and ErbB2. In EGFR, K001MM011 was found to be ranked within the best 10,000 compounds (rank 9527) of the lead-like subset in PDB 3POZ, while, in ErbB2, K001MM011 was ranked not as highly (best rank: 123,665 in PDB 3PP0).

In light of these experimental results and the comparative scarcity of ligands with the intended profiles, we decided to better investigate the kinases involved, with a view towards the possibility to predict the sensibility of a particular target combination.

### 2.4. Kinase Similarities

**Figure 2:**
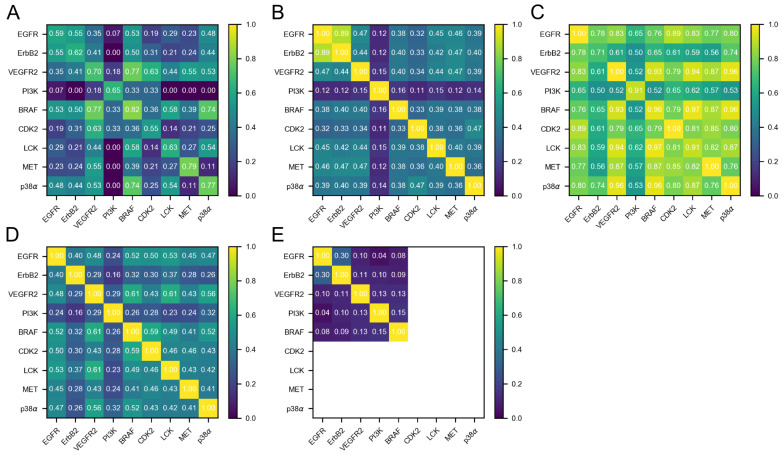
Heat maps of pairwise kinase similarities for the different measures used in this work: (**A**) ligand profile similarity (LigProfSim); (**B**) pocket sequence similarity (PocSeqSim); (**C**) interaction fingerprint similarity (IFPSim); (**D**) pocket structure similarity (PocStrucSim); and (**E**) docking rank similarity (DockRankSim) based on the lead-like subset of ZINC. Note that docking was only performed for the five kinases of Profiles 1–4, thus the remaining entries remain empty (white) in the matrix.

Designing kinase inhibitors with intended dual target activity that avoid binding to one or several specific anti-targets is a non-trivial task, as evidenced by the docking part of our study. To better understand how difficult it may be to design such inhibitors rationally, five different measures of inter-kinase similarity—each contributing a different level of granularity and a different viewpoint—were investigated (Figure 2). Such an analysis potentially enables a priori estimations of the success of these endeavors for a given target/anti-target profile.

#### 2.4.1. Ligand Profile Similarity (LigProfSim)

A first glance at the ChEMBL kinase ligand subsets revealed that none of the investigated kinases seems to be overly selective in terms of the ligands it recognizes, which is in accordance with previous kinome-wide profiling studies [21,27]. Given that the promiscuity values (Table 4, diagonal of Figure 2A and Table S4) range from 0.55 for CDK2 to 0.82 for BRAF, all nine kinases bind more than half of the compounds tested against them at an affinity cut-off of 500 nM. Accordingly, BRAF is the most promiscuous kinase in the set, justifying its use as a general kinase anti-target in this study.

Second, considering LigProfSim, it becomes evident that EGFR, ErbB2 and BRAF are more similar to each other than the remaining kinases (top-left quarter of Figure 2A), which renders finding a compound for Profile 1 (Table 2) a difficult task. With LigProfSim values of 0.53 and 0.55, EGFR is more similar to ErbB2 and BRAF, respectively, than to any other kinase in the set (Table S4). The same holds true for ErbB2, while BRAF has also higher similarities to other kinases in the set. In contrast, with a mean similarity value of 0.18, PI3K has the lowest mean LigProfSim similarity to all nine kinases. This is not unexpected, given that PI3K is the only atypical kinase in the set, but it underlines how challenging the definition of Profile 2 is. Note that, while 4150 compounds were tested against PI3K (with 2706 being active), PI3K has fewer than five common actives with most kinases, except for EGFR (13 common actives of 180 compounds tested against both targets) and VEGFR2 (32 of 175) (see Table 5 and Tables S5 and S6). While all kinases were assayed against at least 1500 compounds, a few other kinase pairs—not including PI3K—exist that have only a low number of tested compounds in common, e.g., CDK2/BRAF (14), CDK2/p38α (8) or ErbB2/p38α (9, see Table S5), which makes thorough comparison difficult. Finally, with a value of 0.35, EGFR and VEGFR2 do not show high similarity from this ligand-centric perspective, while, as mentioned above, VEGFR2 and BRAF show considerably higher similarity (0.77). These numbers indicate that Profile 3 is very difficult.

#### 2.4.2. Pocket Sequence Similarity (PocSeqSim)

Classically, kinases are clustered based on their full sequence similarity, such as in the well-known phylogenetic human kinome tree by Manning et al. [11]. The kinome tree is often considered when checking for relationships among kinases, cross-reactivity and anti-targets. Arguably, EGFR and ErbB2 are the most closely related kinases in the set, both belonging to the TK branch and the EGFR family, followed by similarity to VEGFR2 (TK branch, VEGFR family). BRAF is less closely related (tyrosine-kinase-like [TKL] branch, RAF family). Finally, PI3K belongs to the atypical kinases and is only distantly related. Full kinase details are listed in Table 1.

Here, we refined this sequence-based view of similarity to only consider the 85 residues forming the binding site in each kinase (PocSeqSim). Also in this “pocket sequence” space, the two EGFR family members EGFR and ErbB2 show the highest similarity of 0.89 (Figure 2B, numbers in Table S7). All other kinase pairs have similarity values below 0.48, thus less than 50% identical pocket residues. VEGFR2, MET and LCK, three other kinases from the TK class, have PocSeqSim between 0.42 and 0.47 to EGFR and Erb2; BRAF (TKL), p38α and CDK2 (both from the CMGC family) have values in the range of 0.32 to 0.40. Again, PI3K shows the lowest similarity to all other eight kinases. This indicates that, first, the pocket sequence similarities follow a similar trend as the whole-sequence similarities and, second, that—due to the close relationship of EGFR and ErbB2—other less similar kinases of the TK branch such as VEGFR2, MET and LCK, but also BRAF (TKL), p38α and CDK2 (both from the CMGC family), could be easier-to-satisfy anti-targets of +EGFR+ErbB2 ligands (Figure 2B).

#### 2.4.3. Interaction Fingerprint Similarity (IFPSim)

To take the interplay between the ligand and the protein into account, interaction fingerprint similarities (IFPSim) were investigated. Note that, for each kinase pair, *all* available X-ray structures were compared and that only the similarity between the highest-scoring pair is reported (Figure 2C, numbers in Table S8). In the IFPSim matrix, the diagonal describes the best match among all pairwise IFP comparisons between different structures from the same kinase. Interestingly, ErbB2 has a self-similarity of only 0.71. This could be a consequence of the relatively low structural coverage of this kinase. In fact, ErbB2 is only represented by two structures, whereas, for EGFR, 150 structures are available (Table 5).

With mean similarity values between 0.61 (lowest for PI3K) and 0.83 (highest for VEGFR2), the IFPSim values are generally higher than the LigProfSim and PocSeqSim values described above (Table 4). EGFR has a high mean similarity to all kinases of 0.81, whereas ErbB2 has a lower mean value of 0.64; note again the low structural coverage of ErbB2. While ErbB2 is most similar to EGFR (0.78) with respect to IFPSim (Figure 2C), it is less similar to BRAF (0.65), which would favor the development of a Profile 1 (+EGFR+ErbB2—BRAF) inhibitor. Interestingly, PI3K shows one of the highest similarities to EGFR (0.65), while it is less similar to BRAF (0.52), which, in contrast to other similarity measures, would support the feasibility of designing +EGFR+PI3K—BRAF compounds (Profile 2). In the case of VEGFR2, although similarity to EGFR is high (0.83), we observe an even higher similarity to BRAF (0.93), giving another indication of how difficult it may be to design-out this anti-target. On the other hand, the comparatively high similarity of VEGFR2 to EGFR might give an indication of why our Profile 4 compound actually inhibited EGFR.

#### 2.4.4. Pocket Structure Similarity (PocStrucSim)

Similarities with respect to structural and physicochemical properties of the binding sites were analyzed using the CavBase fast cavity graph comparison algorithm [28,29] (Figure 2D, numbers in Table S9). Note that binding sites were automatically detected using LigSite and thus may vary in precision throughout the different structures, even within the same kinase. Pairwise kinase similarities range from 0.16 (PI3K/ErbB2) to 0.61 (BRAF/VEGFR2 and LCK/VEGFR2) and are—with a mean value of 0.46 over all kinase pairs—generally lower than the IFPSim values described above (Table 4). Interestingly, EGFR and ErbB2 share only moderate similarity in this measure (0.40), while EGFR is more similar to all other kinases (including BRAF; 0.52), except PI3K (0.24). However, it should be noted that the structural coverage for ErbB2 and PI3K is much lower than for the other kinases, with only two structures each (Table 5). Note that EGFR is most similar to the anti-target BRAF (0.52). Thus, according to PocStrucSim, it appears difficult to develop ligands against all multi-target profiles (1–3, Table 2).

#### 2.4.5. Docking Rank Similarity (DockRankSim)

Finally, we leveraged the results of our docking experiments to derive a complementary similarity measure based on the rank correlation of the docked lead-like compounds (Figure 2E). DockRankSim values were calculated using only the top-scoring 25,000 lead-like molecules for each structure (about 0.5% of the ZINC lead-like subset at that time), since control calculations taking into account the entirety of docked molecule sets showed poor discrimination between different kinases. This lack of discrimination is likely due to the fact that the majority of molecules in the lead-like set are not kinase inhibitor-like. Therefore, the docking rank order of molecules past a certain threshold is noisy, i.e., all of them are more or less equally unlikely to bind. However, they will still receive different ranks based on small scoring differences, and these different ranks will lead to rather different—yet meaningless—correlations between the rankings. Only the five kinases that were included in the four docking profiles (Table 2) were considered, i.e., no values for CDK2, LCK, MET and p38α were determined.

EGFR and ErbB2 have by far the highest mutual similarity of 0.3 within this set of kinases and a DockRankSim below 0.12 to all other kinases. While their higher mutual DockRankSim is not surprising given the close relationship between EGFR and ErbB2, it is encouraging that the docking results capture this.

Interestingly, the second highest DockRankSim observed is between PI3K and BRAF (0.15), followed by BRAF and VEGFR2 (0.13) as well as PI3K and VEGFR2 (0.13). This is surprising as PI3K, as atypical kinase, shares a rather low similarity to the remaining kinases using most other measures employed in this study (Figure 2A–D). The remaining DockRankSim values are around 0.1, which seems to be the center of the distribution. The smallest DockRankSim was observed between EGFR and PI3K (0.04), an indication that Profile 2 (+EGFR+PI3K—BRAF) inhibitor design might be a challenge, at least computationally.

#### 2.4.6. Comparison of Similarity Analyses

To shed light on the ease of identifying inhibitors for the respective profiles and the possibility to predict the likelihood that multi-target design endeavors will be successful, five different protein similarity measures were calculated (Figure 2A–E). While the individual relationships between the nine kinases studied differ according to the five measures (which might also be due to missing data or noise in the data, as discussed above), several trends can be observed.

The similarity scores of the PocStrucSim and the IFPSim comparisons are distributed more evenly and clearly correlate with each other (R = 0.78, p<0.001, Figure S2). In addition, the pocket structure- and sequence-based comparisons follow a similar trend (PocStrucSim vs. PocSeqSim R = 0.73, p<0.001). All other pairwise comparisons are less correlated, showing values in the range of R = [0.55, 0.59] with p<0.001 (Figure S2). While several measurements appeared to be correlated, differences between them are not surprising since the measures capture diverse views and thus complementary information of similarity. Nonetheless, it should be noted that the calculated values highly depend on the amount of available data. The conformational space of a kinase might be underrepresented if few kinase structures are available, which affects the structure-related measurements. Furthermore, since ChEMBL only provides a very sparse kinase-compound matrix of experimental measurements, the basis of compounds considered per kinase pair may differ strongly, affecting the LigProfSim values (as well as the promiscuity as defined here).

Besides PocStrucSim, all other measures imply a high similarity between EGFR and ErbB2, which is in favor of +EGFR+ErbB2 inhibitor design. Furthermore, LigProfSim, PocStrucSim and PocSeqSim suggest BRAF as a relevant and frequent anti-target, while this is less clear-cut for the IFPSim and DockRankSim measures. This fact renders design for all three profiles a challenging task. Furthermore, while PI3K is very dissimilar to EGFR from a sequence point of view (cf. Manning tree annotation), it showed higher similarity based on other measures such as IFPSim, which is encouraging for Profile 2 (+EGFR+PI3K—BRAF) design. In this sense, the fact that our docking results did not yield compounds with such a profile would suggest that similarity to the anti-target (in this case, BRAF) larger than to the intended target could be a key factor complicating the detection of the desired compounds.

Overall, our analyses suggest that ligand-, sequence- and structure-based approaches complement each other and can thus yield consistent insights into kinase similarities. It therefore seems advisable to carry out all of these analyses before a (virtual) screening campaign in order to take appropriate steps, e.g., adaptation of the molecule library to be screened, early on. Our ranking comparisons also suggest that similarity between one of the targets and the anti-target that is higher than the similarity between the two intended targets can be used as a prognostic indicator for difficult multi-target profiles.

## 3. Data and Methods

### 3.1. Docking-Based Virtual Screening

Kinase crystal structures that were suitable for docking in general, as well as for the herein discussed purpose in particular, were carefully selected from the Protein Data Bank [14]. Structures were prioritized based on their resolution and the number of missing heavy atoms, with a focus on residues in and around the binding site. Furthermore, structures for target pairs were selected such that the structures for the two kinases involved were as similar as possible. The rationale behind this aim was to maximize the possibility to identify inhibitors binding to both structures. This structural similarity included the overall state of the kinase structure, as determined by the conformation of the DFG and αC motifs, as well as visual comparisons of the binding site residues. Structures with similar side-chain conformations of equivalent amino acids were preferred, as far as such structures existed and the equivalence of amino acids could be rationally established, i.e., for homologous amino acids in EGFR/ErbB2 structure pairs, whereas this was not applicable to, e.g., EGFR/PI3K structure pairs due to their higher dissimilarity. Finally, the crystal structures (PDB IDs given in parentheses) for EGFR (1XKK [30], 3POZ [31]), ErbB2 (3PP0 [31], 3RCD [32]), BRAF (1UWH [33], 3PPK [34]), PI3K (4JPS [35]) and VEGFR2 (2P2H, 3WZD [36]) were downloaded from the PDB (a summary of structural details is presented in Table 6).

The structures were prepared following the protocol in Kolb et al. [37]. Briefly, the first protein chain was used in case several were crystallized. Hydrogens were placed and minimized using the CHARMM (version 31b2) HBUILD command. The ZINC12 [38] lead-like and drug-like subsets (as of July 2015), containing 4.6 and 10.6 M molecules, respectively, were docked into the prepared receptor structures using DOCK 3.6 [39,40,41,42,43] as described in Schmidt et al. [6]. For EGFR, for which a ligand/decoy set is available from DUD-E [44], the prepared structures were additionally validated by their ability to enrich ligands over decoys. AUC values were found to be 0.87 (1XKK) and 0.85 (3POZ), which compares favorably to the value of 0.84 as published by DUD-E [44].

Based on these docking results, compounds were re-scored according to the different selectivity profiles of interest. In our previous work, we introduced a selectivity score for protein pairs, i.e., two docking runs, both being considered as target. Compounds were penalized for unfavorable (i.e., high) ranks in each docking run as well as a high rank difference between these two docking calculations (i.e., good/bad performance in docking A/B; Equation (1) in Schmidt et al. [6]).

Here, this procedure was extended to be applicable to more than two proteins, multiple structures per protein and the proper incorporation of anti-targets. Specifically, the docking calculations for multiple structures of the same kinase (e.g., 1XKK and 3POZ for EGFR) were aggregated by using only the best (i.e., numerically smallest) rank in any of the structures. Second, anti-targets were incorporated by inverting the docking rank order, based on the idea that a good docking performance is disfavored in anti-targets. Third, the equation was extended to multiple proteins by using the average rank (note that ranks for anti-targets were inverted beforehand) in all protein docking calculations of the respective profile (e.g., EGFR, ErbB2 and BRAF) and the rank difference between the highest and lowest docking rank in all proteins. Finally, in contrast to our previous procedure [6], logarithmic ranks were used to focus on the top-scoring molecules, based on the notion that the docking scores (and hence docking ranks) become less discriminating beyond the first few percent of the docked database for very large (and diverse) ligand sets, such as the ones used herein. Altogether, the score *S* of a molecule for the profile comprising kinases 1 to *N* was defined as follows:

(1) $$S_{1,\ldots ,N}=\frac{\frac{1}{N}\sum_{k=1}^{N}P_{k}\;+\;\left(max\left\{P_{1},\ldots ,P_{k},\ldots ,P_{N}\right\}-min\left\{P_{1},\ldots ,P_{k},\ldots ,P_{N}\right\}\right)}{2}$$

with

$$\begin{array}{cc} P_{k} & =log\left(\min_{s}R_{k,s}\right)\\ R_{k,s} & =\frac{r_{k,s}}{m_{k,s}} \end{array}$$

if kinase *k* was defined as target or

$$\begin{array}{cc} P_{k} & =log\left(\max_{s}R_{k,s}\right)\\ R_{k,s} & =1-\frac{r_{k,s}}{m_{k,s}} \end{array}$$

if kinase *k* was defined as anti-target. Here, $P_{k}$ denotes the rank of a compound in kinase *k* aggregated over all structures *s* of this kinase. $R_{k,s}$ denotes the scaled docking rank of the compound, calculated from the nominal docking rank $r_{k,s}$ of this compound and the total number of molecules $m_{k,s}$ that were docked into the *s*th structure of the *k*th kinase.

The poses of molecules receiving top ranks after applying this rescoring were visually inspected in their respective protein structure. This inspection is necessary in order to remove compounds which are ranked favorably for the wrong reasons, i.e., because of deficiencies in present-day force fields. Examples are unsatisfied hydrogen bond donors; burial of polar protein residues through apolar ligand moieties; charge mismatches; and ligand conformations with high strain.

### 3.2. Experimental Testing

#### 3.2.1. DiscoverX KINOMEscan

Ligand binding experiments for the molecules selected from Profiles 1 and 2 towards nine kinases (EGFR, ErbB2, LCK, CDK2, BRAF, MET, p38α, PI3K and VEGFR2) and for molecules selected from Profiles 3 and 4 towards four kinases (EGFR, ErbB2, BRAF and VEGFR2) were carried out by DiscoverX using the supplied protocol as described in the Supplementary Materials. Briefly, ligand affinity was measured by competition with a resin-bound standard ligand and washed-off kinase concentration was determined via qPCR.

Summarizing, binding of a compound to a kinase was tested in comparison to a control compound (see Table S2). Lower values generally indicate a higher affinity of the compound to the protein with values below 35% being considered as significant binding according to the information of DiscoverX.

#### 3.2.2. Eurofins In Vitro Assay

Kinase inhibition assays for EGFR, ErbB2, PI3K and BRAF were carried out by Eurofins Cerep following the protocols of Weber et al. [45] (EGFR), Quian et al. [46] (ErbB2), Sinnamon et al. [47] (PI3K) and Kupcho et al. [48] (BRAF). Briefly, except for PI3K, compounds were incubated with the respective kinase, ATP, and a substrate analog, and the effect of each compound on phosphorylation was measured. In the case of PI3K, the displacement of biotinylated PIP3 from a PIP3-binding complex by unlabelled PIP3 (produced from PIP2 by PI3K) was measured by Homogeneous Time Resolved Fluorescence (HTRF).

Finally, inhibition of the respective kinases is calculated as the percentage inhibition of control activity. According to Eurofins, values above 50% inhibition represent significant inhibition and values between 25% and 50% weak inhibitory effects (Table S3).

#### 3.2.3. IC${}_{50}$ Determination

IC${}_{50}$ determinations for EGFR, its mutants and ErbB2-insYVMA (Carna Biosciences, lot13CBS-0005K for EGFR-wt; Carna, lot13CBS-0537B for EGFR-L858R; Carna, lot12CBS-0765B for EGFR-L858R/T790M; and ProQinase, lot1525-0000-1/003 for ErbB2-insYVMA) were performed with the HTRF KinEASE-TK assay from Cisbio according to the manufacturer’s instructions. Briefly, the amount of kinase in each reaction well was set to 0.60 ng EGFR-wt (0.67 nM), 0.10 ng EGFR-L858R (0.11 nM), 0.07 ng EGFR-T790M/L858R (0.08 nM), or 0.01 ng ErbB2-insYVMA (0.01 nM). An artificial substrate peptide (TK-substrate from Cisbio) was phosphorylated by EGFR or ErbB2. After completion of the reaction (reaction times: 25 min for EGFR-wt, 15 min for L858R, 20 min for L858R/T790M, and 40 min for ErbB2-insYVMA), the reaction was stopped by addition of buffer containing EDTA as well as an anti-phosphotyrosine antibody labeled with europium cryptate and streptavidin labeled with the fluorophore XL665. FRET between europium cryptate and XL665 was measured after an additional hour of incubation to quantify the phosphorylation of the substrate peptide. ATP concentrations were set at their respective $K_{m}$-values (9.5 µM for EGFR-wt, 9 µM for L858R, 4 µM for L858R/T790M and 6 µM for ErbB2-insYVMA) while a substrate concentration of 1 µM, 225 nM, 200 nM and 1 µM was used. Kinase and inhibitor were preincubated for 30 min before the reaction was started by addition of ATP and substrate peptide. An EnVision multimode plate reader (Perkin Elmer) was used to measure the fluorescence of the samples at 620 nm (Eu${}^{3+}$-labeled antibody) and 665 nm (XL665-labeled streptavidin) 50 µs after excitation at 320 nm. The quotient of both intensities for reactions made with eight different inhibitor concentrations was then analyzed using the Quattro Software Suite for IC${}_{50}$-determination. Each reaction was performed in duplicate, and at least three independent determinations of each IC${}_{50}$ were made.

### 3.3. Kinase Similarity Measures

The nine protein kinases investigated in this study were compared with five measures: their ligand binding profiles (LigProfSim), pocket sequence (PocSeqSim), interaction fingerprint (IFPSim) and structural information (PocStrucSim), as well as docking ranks (DockRankSim).

#### 3.3.1. Ligand Profile Similarity (LigProfSim)

To compare kinases from a ligand point of view, their similarity with respect to binding the same ligands was investigated. The kinase subset of ChEMBL v.27 [49] was used as the profiling dataset, assembled from https://github.com/openkinome/kinodata/releases/tag/_pub_ligprofsim (accessed September 2020). Only compounds measured in binding assays yielding a standard activity value as IC${}_{50}$ were taken into account. If the same compound was measured several times in the same assay (against the same kinase), only the lowest IC${}_{50}$ value was kept (most active). Compounds were considered active against a kinase if their IC${}_{50}$ value was below 500 nM, otherwise inactive. For each of the nine kinases studied here, the total number of measured compounds and the number of active compounds was determined (Table 5).

The pairwise ligand profile similarity (LigProfSim) between two kinases was calculated as the ratio of compounds active on both kinases divided by the total number of compounds tested on both kinases (Figure 2A, absolute values in Tables S4–S6). Note that, for the individual kinases, this “self-similarity” yields the fraction of active compounds with respect to all compounds tested, which can also be interpreted as a simple measure for promiscuity (Table 4).

#### 3.3.2. Pocket Sequence Similarity (PocSeqSim)

Pocket sequences and binding site definitions were taken from the KLIFS database [15,16,17]. Based on the analysis of known kinase–ligand crystal structures, van Linden et al. [15] defined the ATP-binding pocket of kinases by 85 residues which cover most interactions with known inhibitors (front and back-cleft binders). These residues include known motifs such as the DFG motif, the hinge region and the αC-helix.

To compare kinase binding sites based on sequences, the master multiple sequence alignment (MSA) of the 85 binding pocket residues for all human kinases available from KLIFS was used and the nine kinases investigated in this work were extracted. Pocket sequence similarity (PocSeqSim)—in this case residue identity—between two kinases was computed by comparing the residues at each of the 85 positions. Thus, the PocSeqSim for two binding site sequences equals the ratio of identical residues within the fixed length MSA of 85 positions. The score ranges from 0 to 1, where 0 indicates no identical residues and 1 indicates complete identity (Table S7).

#### 3.3.3. Interaction Fingerprint Similarity (IFPSim)

All DFG-in and DFG-out structures for the nine human kinases under investigation, namely EGFR, ErbB2, PI3K, MET, CDK2, BRAF, p38α, LCK and VEGFR2, were fetched from the KLIFS database with https://github.com/volkamerlab/opencadd, which uses the KLIFS Swagger API [17]. This query yielded 2091 structures (as of 27 July 2020). Only structures with orthosteric ligands were kept (1817 structures). For many kinases, several PDB structures are available and many structures contain more than one chain (and occasionally also alternative models), which are provided as separate entries in KLIFS. Whenever one structure was represented by more than one chain/alternative model entry, only the entry with the highest KLIFS quality score [16] was selected (if two had the same quality, the first one was kept arbitrarily). The quality score describes the alignment and structure quality ranging from 0 (bad) to 10 (flawless). This yielded a filtered set of 965 kinase structures (numbers per kinase in Table 5). For every structure, KLIFS provides information on the kinase–ligand interaction stored in an Interaction FingerPrint (IFP). The IFP encodes seven different interaction types (hydrophobic contact, aromatic face-to-face, aromatic edge-to-face, H-bond donor–acceptor, H-bond acceptor–donor, ionic positive–negative and ionic negative–positive) that can potentially be formed between each of the 85 pocket residues and the respective ligand in a bit string as either present (1) or absent (0) [15,16]. The Tanimoto similarity between every IFP pair of the 965 structures was calculated, resulting in multiple structure-pair comparisons for each kinase pair. Finally, a reduced matrix of size 9×9 was produced in which for each kinase pair only the highest IFP similarity (IFPSim) score among all structure-pair scores was stored (Table S8).

#### 3.3.4. Pocket Structure Similarity (PocStrucSim)

For the particular set of kinases investigated here, a set of 183 different PDB structures was compiled manually using the KLIFS dataset and a set of structures that has initially been considered for the docking screens. The manual selection was focused on choosing those kinase structures that featured similar binding sites to EGFR/ErbB2 and high structural quality (such as high resolution and few missing residues), also considering the correlation coefficient of the docking ranks. Furthermore, DFG-in and DFG-out structures were included to allow for diversity. After downloading the structures from the PDB, the files were processed with the API-RP package in the CSD Enterprise suite 2018 by CCDC, detecting all cavities using LigSite [50,51]. The predicted set of 909 cavities for 181 structures was further reduced by filtering for cavities containing at least one orthosteric ligand, resulting in 248 cavities from 176 different structures. It should be noted that some of these cavities emerged from different chains of the same structure and, therefore, contained the same ligand. Although the number of structures was decreased during this process, we made sure that at least two different structures for each kinase were still present in the final cavity set (Table 5). Furthermore, the set contained cavities for each of the structures used during the docking calculations, except for the structure with PDB ID 4JPS (PI3K), for which LigSite was not able to detect the correct cavity.

Each of the remaining cavities was then compared to all other cavities using the fast graph comparison method by CCDC [29]. In brief, the binding pocket is described by a graph model based on a set of pseudocenters with assigned surface patches containing information about the properties of the surrounding amino acids. In addition to the original CavBase implementation, the new method includes convexity and concavity measures in the pseudocenters as shape representation. Finally, two binding pockets were compared using a clique detection algorithm which was improved from the original CavBase algorithm [28,29]. Last, as for the IFPSim measure, the maximum similarity over all structure comparisons per kinase pair is reported.

#### 3.3.5. Docking Rank Similarity (DockRankSim)

The docking rank similarity was calculated based on the notion that similar structures enrich similar ligands in the docking process. The similarity between two docking runs, each targeting a certain structure, was quantified by calculating the Spearman rank correlation of the common molecule set of the top-scoring molecules of both dockings. More precisely, to calculate the DockRankSim between two dockings, the top-ranked 25,000 molecules in both dockings were taken and the molecules common to both sets identified. For the calculation of the DockRankSim, only the dockings of the ZINC lead-like subset were considered. For this intersection, the ranks of the molecules were renumbered and the Spearman rank correlation was calculated. We restricted the calculation of the rank correlation to the top-scoring molecules, as we found this to lead to more discriminating DockRankSim values (data for full set not shown). A cutoff of 25,000 was identified to yield relevant results. However, it must be noted that this cutoff was not systematically optimized to yield the largest possible spread in DockRankSim values. The values calculated in this way describe how similar the compound ranking between two docking runs, i.e., two protein structures, is. To compare kinases instead of structures, we used the maximum observed DockRankSim of all pairwise structure comparisons between the respective two kinases.

## 4. Conclusions

In this study, we investigated parallel docking to disease-relevant kinase profiles, combining two targets and one anti-target. The choice of the initial profile was guided by biology: dual inhibitors of EGFR and ErbB2 are regarded as an advantageous treatment option for several carcinomas, whereas BRAF is a common undesired anti-target.

While being biologically meaningful, this profile is also a challenging test case of the precision of docking calculations, given the mutual similarity of the ATP binding site of the three kinases. Nonetheless, we were able to identify one ligand with the desired profile, namely compound DS39984 against Profile 1, with IC${}_{50}$ values on the targets below 324 nM. This is very close to the expectation value assuming a hit rate of approximately 10–25% (0.25×0.25×0.90=0.056) and a selection of 18 molecules from the docking calculations.

We then compared this with another profile combination, +EGFR+PI3K—BRAF (Profile 2), and at the same time investigated whether the likelihood for success (i.e., finding a ligand that fulfils the profile) can be predicted based on data derived from the protein structures. The profile +EGFR+PI3K—BRAF turned out to be hard to find a ligand for, and this was also reflected in the kinase similarity metrics (Figure 2). Finally, we tested a profile including EGFR and VEGFR2 as targets, due to the interest in them for cancer treatment, and tried again to design out binding to BRAF. As in the case of +EGFR+PI3K—BRAF, the higher similarity of VEGFR2 to BRAF (compared to EGFR) in most measures can be a hint why this docking did not yield the desired results. An alternative option, which would agree with the lack of positive results in the single docking performed for the target VEGFR2, is to select alternative starting structures, if available, or a different ligand database to further explore this profile.

Based on our findings and the further investigations into different similarity measures of kinases, several conclusions about the factors that determine the likelihood of successful predictions in multi-target settings can be drawn. First, for the present set of kinases, the various measures we calculated in this work largely agree with respect to which kinases are more similar to each other. This is important, because it means that, for a first estimate, one can go with a measure that can be computed in a fast and computationally inexpensive way and already get a largely correct view of the relationship of the targets involved. It also means that the ligand-centric and protein-centric views of ligand–protein interactions match to quite some degree.

Second, we only managed to pick few compounds from the docking runs, because few potential hits with plausible binding modes were identified in the top ranks of the combined scoring. Naturally, this means that the results for several of the profiles need to be interpreted with caution, as the numbers of data points are small. However, even if we had picked more compounds from lower ranks, the vast majority of them would likely have been inactive, as docking in general is able to prioritize ligands over nonbinders [52].

Third, the docking rank correlation of the top-ranked poses is very low (Figure 2E), which indicates that there exists only a limited number of substances in chemical databases for a given kinase profile. This lends additional support to docking strategies using (ultra-)large libraries of virtual compounds, as having access to larger and more diverse fractions of chemical space is certainly beneficial [52,53]. It has to be noted, however, that a certain amount of the rank correlation difference might also stem from the use of rigid protein structures in docking.

In conclusion, while docking to identify ligands gets progressively harder with more and more elaborate profiles composed of targets and anti-targets, one can try to estimate the chances of success already from protein-structure-, protein-sequence- and ligand-space-based methods. This is encouraging in the sense that protein and ligand space show a certain amount of congruence, i.e., that kinases that are close in structure or sequence space also recognize similar ligands, and supports the ongoing efforts to computationally expand chemical space to search for kinase inhibitors with tailored binding profiles.

## Figures and Tables

**Table 1:** List of kinases used in this study.

Kinase ${}^{a}$	Synonyms	UniProt ID	Group	Family
EGFR	ErbB1	P00533	TK	EGFR
ErbB2	Her2	P04626	TK	EGFR
PI3K	PI3KCA, p110	P42336	Atypical	PIK
VEGFR2	KDR	P35968	TK	VEGFR
BRAF	-	P15056	TKL	RAF
CDK2	-	P24941	CMGC	CDK
LCK	-	P06239	TK	Src
MET	-	P08581	TK	MET
p38	MAPK14	Q16539	CMGC	MAPK

*^a^* EGFR, epidermal growth factor receptor; ErbB2, Erythroblastic leukemia viral oncogene homolog 2; PI3K, phosphatidylinositol-3-kinase; VEGFR2, vascular endothelial growth factor receptor 2; BRAF, rapidly accelerated fibrosarcoma isoform B; CDK2, cyclic-dependent kinase 2; LCK, lymphocyte-specific protein tyrosine kinase; MET, mesenchymal-epithelial transition factor; p38a, p38 mitogen activated protein kinase a.

**Table 2:** Definitions of kinase profiles and the numbers of screening compounds selected for each profile.

ID	Kinase Profile ${}^{a}$	No. of Tested Compounds
1	+EGFR+ErbB2—BRAF	18 ${}^{b,c}$
2	+EGFR+PI3K—BRAF	9 ${}^{b}$
3	+EGFR+VEGFR2—BRAF	8 ${}^{c}$
4	+VEGFR2	4

*^a^* + and −indicate targets and anti-targets, respectively. *^b^* Three compounds are identical between Profiles 1 and 2 but were independently selected from the docking calculations against both profiles. *^c^* One compound is identical between Profiles 1 and 3 but was independently selected from the docking calculations against both profiles.

**Table 3:** Assay results for identified hit molecules.

Compound	P ${}^{a}$	Research Lab	Unit	EGFR	ErbB2	ErbB2insYVMA	BRAF
DS39984	1	DiscoverX	% ctrl. activity at 10 µM	17	21	n.d. ${}^{c}$	– ${}^{d}$
DS39984	1	Eurofins	% inhib. at 20 µM ± s.d.	59±3.2	– ${}^{b}$	n.d. ${}^{c}$	– ${}^{b}$
DS39984	1	Rauh Lab	IC${}_{50}$ ± s.d.	324±50 nM	n.d. ${}^{c}$	220±3 nM	n.d. ${}^{c}$
K001MM011	4	DiscoverX	% ctrl. activity at 10 µM	1.4	53	n.d. ${}^{c}$	– ${}^{d}$

*^a^* + Profile as per Table 2; *^b^* Below 50% cutoff for hit as recommended by Eurofins; *^c^* not determined; *^d^* percent control activity ≥ 99%, i.e., no measurable inhibition.

**Table 4:** Kinase promiscuity measures calculated as the ratio of ligands active on a specific kinase (column 2). In Columns 3–6, mean values and standard deviations (s.d.) of ligand profile similarity (LigProfSim), pocket sequence similarity (PocSeqSim), interaction fingerprint similarity (IFPSim) and pocket structure similarity (PocStrucSim) per kinase are given. Note: Two kinases having a similar mean value for a particular similarity measurement does not imply that they are similar to each other (especially when large s.d. values are associated with the measure; see Figure 2 for pairwise kinase comparisons).

Kinase	Promiscuity ${}^{a}$	Mean (±s.d.) ${}^{b}$			
		LigProfSim	PocSeqSim	IFPSim	PocStrucSim
EGFR	0.59	0.37 (±0.18)	0.50 (±0.28)	0.81 (±0.10)	0.50 (±0.21)
ErbB2	0.62	0.37 (±0.19)	0.50 (±0.28)	0.64 (±0.09)	0.38 (±0.24)
PI3K	0.65	0.18 (±0.23)	0.23 (±0.29)	0.61 (±0.13)	0.33 (±0.26)
BRAF	0.82	0.56 (±0.18)	0.42 (±0.23)	0.82 (±0.16)	0.52 (±0.21)
CDK2	0.55	0.33 (±0.17)	0.40 (±0.24)	0.80 (±0.12)	0.49 (±0.21)
LCK	0.63	0.34 (±0.22)	0.45 (±0.23)	0.82 (±0.13)	0.50 (±0.21)
MET	0.79	0.31 (±0.24)	0.45 (±0.23)	0.79 (±0.14)	0.46 (±0.22)
p38	0.77	0.43 (±0.27)	0.44 (±0.23)	0.82 (±0.15)	0.47 (±0.21)
VEGFR2	0.70	0.51 (±0.18)	0.46 (±0.23)	0.83 (±0.16)	0.50 (±0.23)

*^a^* Kinase promiscuity based on ligand affinity data from ChEMBL, measured as ratio of active compounds over tested compounds (activity threshold IC50 = 500 nM, cf. Table 5); *^b^* mean ± standard deviation (s.d.) values for LigProfSim, PocSeqSim, IFPSim and PocStrucSim of the respective kinase to all nine kinases (including itself) in the set.

**Table 5:** Dataset composition for the similarity analysis. Listed are the numbers of compounds active and tested against each kinase used for the ligand profile similarity (LigProfSim), as well as number of structures used for the interaction fingerprint similarity (IFPSim) and the pocket structure similarity (PocStrucSim) calculations.

Kinase (Family/Group)	# Compounds		# Structures	
	Actives	Tested	IFPSim	PocStrucSim
EGFR (TK/EGFR)	3382	5702	150	15
ErbB2 (TK/EGFR)	1048	1690	2	2
PI3K (Atypical/PIK)	2706	4150	26	2
VEGFR2 (TK/VEGFR)	5197	7426	41	13
BRAF (TKL/RAF)	2968	3625	69	25
CDK2 (CMGC/CDK)	837	1520	377	43
LCK (TK/Src)	976	1552	34	29
MET (TK(MET))	2248	2851	70	11
p38(CMGC/MAPK)	2753	3581	196	36
Total	22,115	32,097	965	176

**Table 6:** Kinase structures used in docking experiments.

Kinase	PDB	DFG ${}^{a}$	αC ${}^{b}$
EGFR	1XKK	in	out
EGFR	3POZ	in	out
ErbB2	3PP0	in	in
ErbB2	3RCD	in	out-like
BRAF	1UWH	out	out
BRAF	3PPK	in	in
PI3K	4JPS	in	in
VEGFR2	2P2H	in	out
VEGFR2	3WZD	in	out

*^a^* Orientation of the conserved DFG motif (in/out), annotation from KLIFS [12]; *^b^* conformation of the a αC-helix (in/out), annotation from KLIFS [12].

## Data Availability

Raw data files of docking and similarity calculations are available from the corresponding authors upon reasonable request.

## Supplementary Materials

The following are available online. Figure S1: Docking poses of ligand DS39984 bound to BRAF structures, Figure S2: Comparison of different similarity measures for pairwise kinase structure comparisons, Table S1: IDs and 2D depictions of all compounds tested in the different kinase assays as well as the docking profile they were selected from, Table S2: Experimental % control values from the DiscoverX kinase assay, Table S3: Experimental results from the Eurofins assay, Table S4: LigProfSim: Pairwise similarity matrix, Table S5: LigProfSim counts: Number of ChEMBL compounds commonly tested in each kinase pair, Table S6: LigProfSim common actives: Number of ChEMBL compounds commonly active in each kinase pair, Table S7: PocSeqSim: Pairwise similarity matrix, Table S8: IFPSim: Pairwise similarity matrix, Table S9: PocStrucSim: Pairwise similarity matrix.
