# Supplementary material for: Analyzing Kinase Similarity in Small Molecule and Protein Structural Space to Explore the Limits of Multi-Target Screening
Source: Molecules. 2021 Jan 26;26(3):629. doi: 10.3390/molecules26030629 (PMC7865522; doi:10.3390/molecules26030629)
Supplement: Supplementary file 1 [file molecules-26-00629-s001.pdf]

Supplementary Materials: Analyzing kinase similarity in small molecule and protein structural space to explore the limits of multi-target screening

Denis Schmidt, Magdalena M. Scharf, Dominique Sydow, Eva Aßmann, Maria Martí-Solano, Marina Keul, Andrea Volkamer and Peter Kolb

1. Supplementary Methods

1.1. DiscoverX kinase assay

For most assays, kinase-tagged T7 phage strains were grown in parallel in 24-well blocks in an *E.coli* host derived from the BL21 strain. *E.coli* were grown to log-phase and infected with T7 phage from a frozen stock (multiplicity of infection = 0.4) and incubated with shaking at 32°C until lysis (90-150 minutes). The lysates were centrifuged (6,000 x g) and filtered (0.2μm) to remove cell debris. The remaining kinases were produced in HEK-293 cells and subsequently tagged with DNA for qPCR detection. Streptavidin-coated magnetic beads were treated with biotinylated small molecule ligands for 30 minutes at room temperature to generate affinity resins for the kinases. The liganded beads were blocked with excess biotin and washed with blocking buffer (SeaBlock [Pierce], 1% BSA, 0.05% Tween 20, 1 mM DTT) to remove unbound ligands and to reduce non-specific phage binding. Binding reactions were assembled by combining kinases, liganded affinity beads, and test compounds in 1x binding buffer (20% SeaBlock, 0.17x PBS, 0.05% Tween 20, 6 mM DTT). Test compounds were prepared as 40x stocks in 100% DMSO and directly diluted into the assay. All reactions were performed in polypropylene 384-well plates in a final volume of 0.02 ml. The assay plates were incubated at room temperature with shaking for 1 hour and the affinity beads were washed with wash buffer (1x PBS, 0.05% Tween 20). The beads were then re-suspended in elution buffer (1x PBS, 0.05% Tween 20, 0.5 μM non-biotinylated affinity ligand) and incubated at room temperature with shaking for 30 minutes. The kinase concentration in the eluates was measured by qPCR.

2. Supplementary Figures

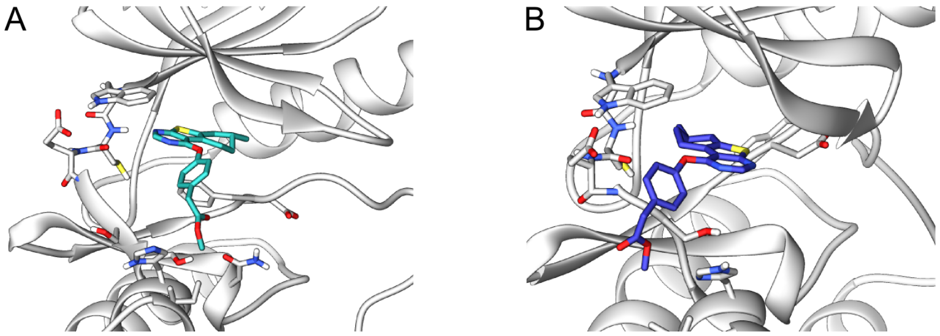

Figure S1. Structure of ligand DS39984 bound to the BRAF structures PDB 1UWH, DFG-out (A) and PDB 3PPK, DFG-in (B). The protein structure is shown as cartoon, colored in grey. The compound and interacting binding site residues are represented as sticks.

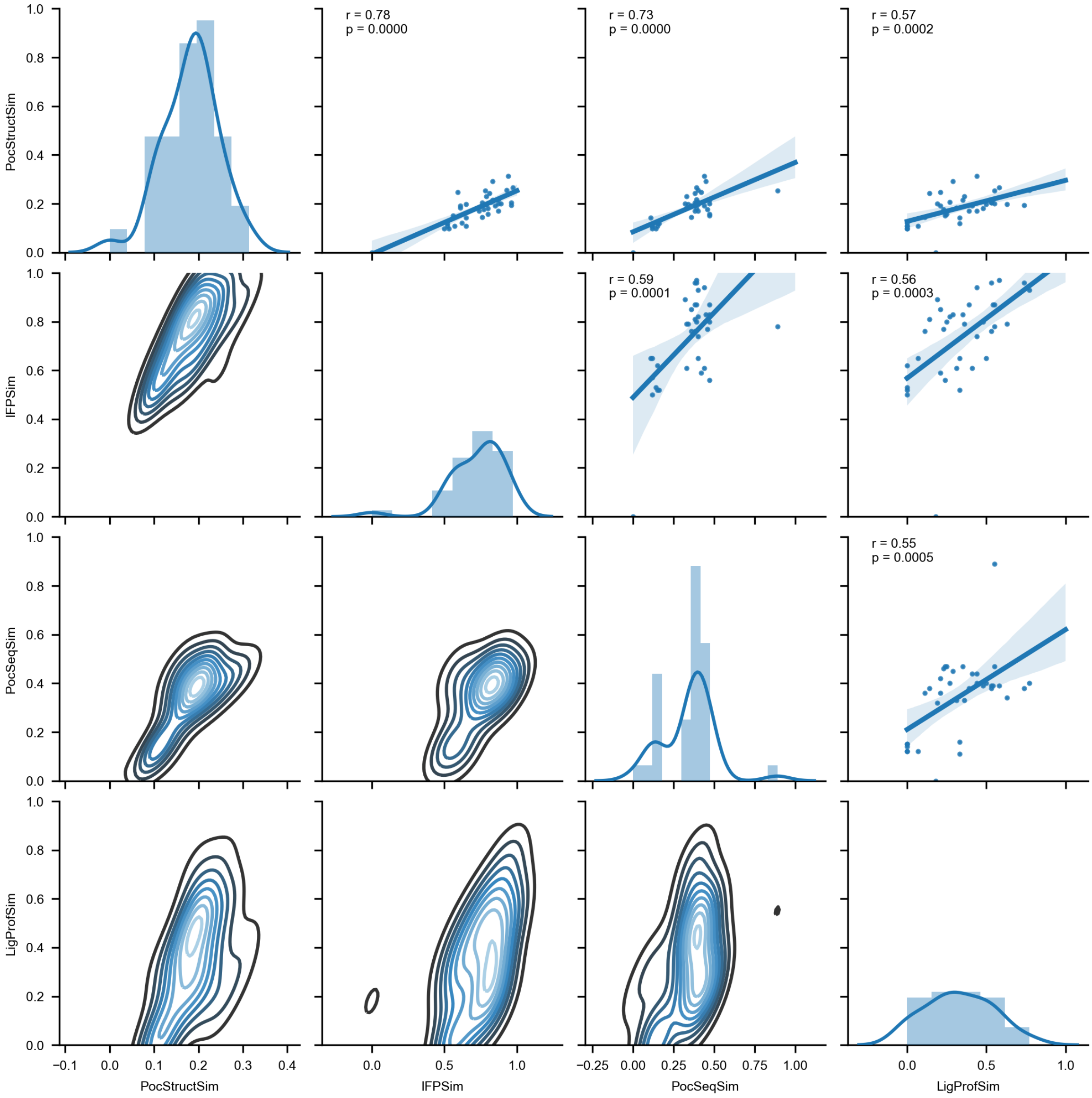

Figure S2. Comparison of different similarity measures for pairwise kinase structure comparisons. Diagonal: Distributions of structure similarities for the herein described similarity measures. Lower triangular matrix: Bivariate distributions of similarities per pairs of similarity measures, shown as isocontours with light blue indicating high densities and dark blue indicating low densities. Upper triangular matrix: Scatter plots of similarities per pairs of similarity measures with fitted regression lines (dark lines) and 95% CI intervals of regression (light blue shades)

3. Supplementary Tables

3.1. Compound lists and experimental results

Table S1. IDs and 2D depictions of all compounds tested in the different kinase assays as well as the docking profile they were selected from. Three compounds were selected independently from two different profiles and are marked accordingly.

Mol ID	ZINC ID	2D structure	Profile
Actives			
DS39984	C03283998	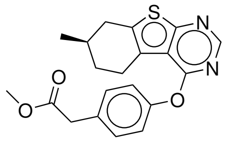	+EGFR+ErbB2-BRAF
K001MM011	C32808493	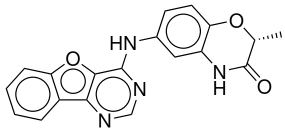	+VEGFR2
Inactives			
DS04644	C84640464	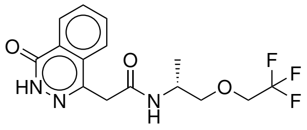	+EGFR+ErbB2-BRAF
DS05168	C04940516	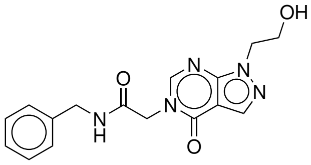	+EGFR+ErbB2-BRAF
DS18339	C71281833	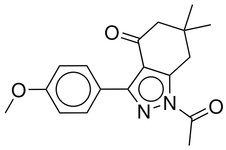	+EGFR+ErbB2-BRAF
DS34376	C95373437	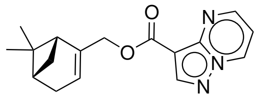	+EGFR+ErbB2-BRAF
DS44245	C47934424	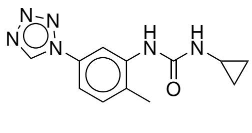	+EGFR+ErbB2-BRAF
DS44738[b]	C48954473	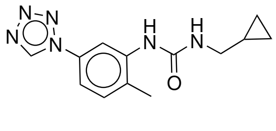	+EGFR+ErbB2-BRAF

DS57124	C17055712	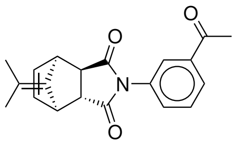	+EGFR+ErbB2-BRAF
DS59212	C09205921	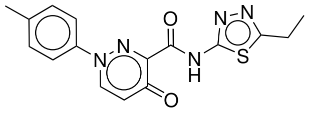	+EGFR+ErbB2-BRAF
DS72975	C06807297	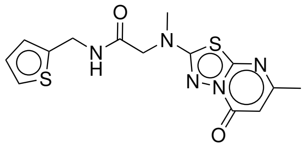	+EGFR+ErbB2-BRAF
DS74417	C95387441	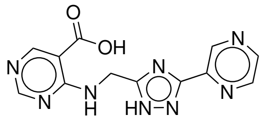	+EGFR+ErbB2-BRAF
DS75739	C48697573	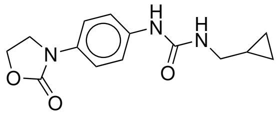	+EGFR+ErbB2-BRAF
DS76514	C00137651	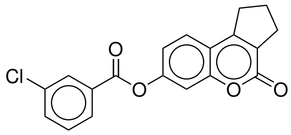	+EGFR+ErbB2-BRAF
DS84326	C20858432	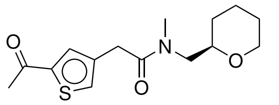	+EGFR+ErbB2-BRAF
DS99367	C71899936	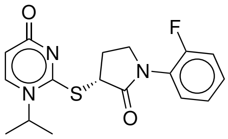	+EGFR+ErbB2-BRAF
DS23815	C71422381	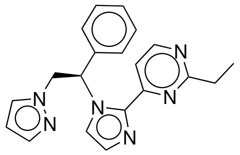	+EGFR+PI3K-BRAF
DS31939	C02343193	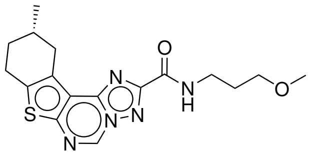	+EGFR+PI3K-BRAF

DS52225	C44425222	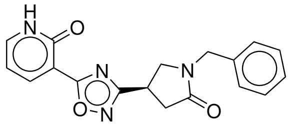	+EGFR+PI3K-BRAF
DS62156	C08706215	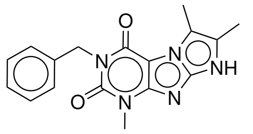	+EGFR+PI3K-BRAF
DS74631	C07397463	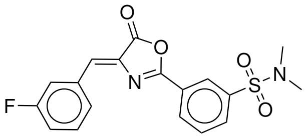	+EGFR+PI3K-BRAF
DS82066	C02228206	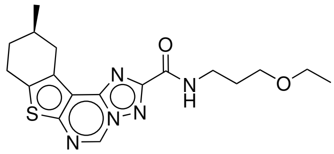	+EGFR+PI3K-BRAF
DS11689[a]	C02341168	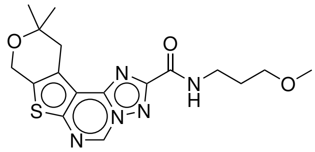	+EGFR+PI3K-BRAF/ +EGFR+ErbB2-BRAF
DS66846[a]	C02226684	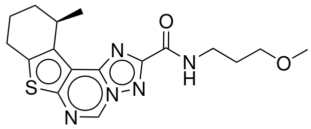	+EGFR+PI3K-BRAF/ +EGFR+ErbB2-BRAF
DS74871[a]	C07397487	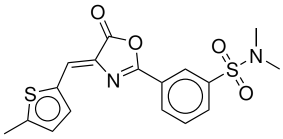	+EGFR+PI3K-BRAF/ +EGFR+ErbB2-BRAF
K001MM002	C97100024	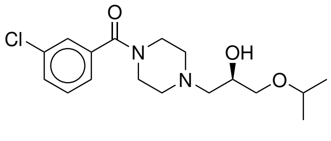	+EGFR+VEGFR2-BRAF
K001MM003	C04266692	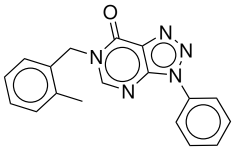	+EGFR+VEGFR2-BRAF
K001MM004	C48922370	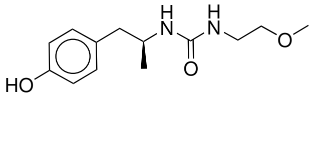	+EGFR+VEGFR2-BRAF

K001MM005	C76064467	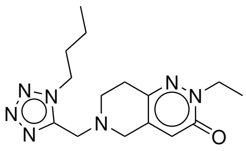	+EGFR+VEGFR2-BRAF
K001MM006	C96153842	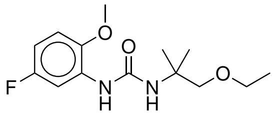	+EGFR+VEGFR2-BRAF
K001MM007	C97142813	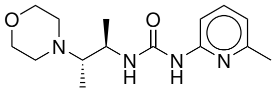	+EGFR+VEGFR2-BRAF
K001MM008	C40067740	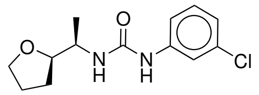	+EGFR+VEGFR2-BRAF
K001MM009[b]	C48954473	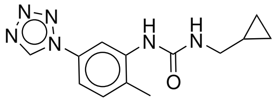	+EGFR+VEGFR2-BRAF
K001MM010	C96160364	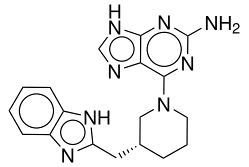	+VEGFR2
K001MM012	C03453350	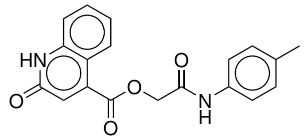	+VEGFR2
K001MM013	C23551796	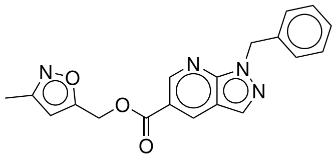	+VEGFR2

[a] Compounds were selected independently from docking campaigns against two profiles.
[b] Compound was selected independently from two docking profiles and tested twice in separate test rounds during experimental validation.

Table S2. Experimental % control values from the DiscoverX kinase assay. Compounds were tested against the nine kinases EGFR, ErbB2, LCK, CDK2, BRAF, MET, p38α, PI3K and VEGFR2, unless otherwise stated. Binding of kinase and compound were tested at a compound concentration of 10 µM and in comparison to a control compound. Lower values indicate a higher affinity of the compound to the protein and values below 35% indicate significant binding according to information of the CRO.

Mol ID	ZINC ID	BRAF	EGFR	ErbB2	VEGFR2	CDK2	LCK	MET	p38α	PI3K
Actives										
DS39984	C03283998	99	17	21	99	100	89	100	100	100
K001MM011[a]	C32808493	100	1.4	53	99	n.t.	n.t.	n.t.	n.t.	n.t.
Inactives										
DS04644	C84640464	97	100	100	100	99	96	100	100	100
DS05168	C04940516	98	100	100	100	99	95	94	100	100
DS18339	C71281833	100	97	100	97	100	89	100	99	100
DS34376	C95373437	92	90	100	100	99	100	100	100	100
DS44245	C47934424	99	100	100	98	100	100	90	100	100
DS44738[b]	C48954473	100	100	91	100	100	100	84	100	94
DS57124	C17055712	100	100	91	100	100	100	94	100	100
DS59212	C09205921	100	100	100	100	100	100	97	99	87
DS72975	C06807297	100	96	87	99	100	100	78	100	100
DS74417	C95387441	86	89	100	100	100	100	100	100	97
DS75739	C48697573	100	100	94	100	100	100	85	87	100
DS76514	C00137651	89	87	96	100	100	100	90	97	100
DS84326	C20858432	100	100	97	100	100	100	95	90	100
DS99367	C71899936	100	93	100	100	100	100	87	91	100
DS23815	C71422381	99	100	100	100	99	100	99	100	97
DS31939	C02343193	100	100	100	100	100	99	100	100	73
DS52225	C44425222	100	100	94	98	100	100	91	100	100
DS62156	C08706215	95	90	96	100	100	100	87	97	66
DS74631	C07397463	100	97	95	100	100	87	100	83	100
DS82066	C02228206	83	92	90	100	100	100	100	93	89
DS11689	C02341168	88	100	99	100	100	100	99	100	99
DS66846	C02226684	100	100	96	100	100	100	92	100	100
DS74871	C07397487	100	100	100	100	100	90	95	81	100
K001MM002[a]	C97100024	89	97	92	100	n.t.	n.t.	n.t.	n.t.	n.t.
K001MM003[a]	C04266692	95	99	99	100	n.t.	n.t.	n.t.	n.t.	n.t.
K001MM004[a]	C48922370	100	100	100	100	n.t.	n.t.	n.t.	n.t.	n.t.
K001MM005[a]	C76064467	99	100	100	100	n.t.	n.t.	n.t.	n.t.	n.t.
K001MM006[a]	C96153842	100	96	100	100	n.t.	n.t.	n.t.	n.t.	n.t.
K001MM007[a]	C97142813	87	90	94	100	n.t.	n.t.	n.t.	n.t.	n.t.
K001MM008[a]	C40067740	100	100	100	100	n.t.	n.t.	n.t.	n.t.	n.t.
K001MM009[a,b]	C48954473	96	100	98	98	n.t.	n.t.	n.t.	n.t.	n.t.
K001MM010[a]	C96160364	100	95	90	97	n.t.	n.t.	n.t.	n.t.	n.t.
K001MM012[a]	C03453350	100	90	100	91	n.t.	n.t.	n.t.	n.t.	n.t.
K001MM013[a]	C23551796	100	100	94	90	n.t.	n.t.	n.t.	n.t.	n.t.

[a] Compounds were only tested against four kinases (EGFR, ErbB2, BRAF and VEGFR2).
[b] Compound was selected independently from two docking profiles and tested separately during experimental validation.
n.t.: not tested.

Table S3. Experimental results from the Eurofins assay. Inhibition of four kinases (EGFR, PI3K, ErbB2, BRAF) was measured at compound concentrations of 20 µM. Inhibition was calculated as % inhibition of control activity. According to CRO, values above 50% inhibition represent significant inhibition, values above 25% weak inhibition effect and values below 25% as well as negative values are usually not significant. Results are reported in *mean (SD)* format.

Mol ID	ZINC ID	EGFR	PI3K	ErbB2	BRAF
Actives					
DS39984	C03283998	58.9 (3.2)	-6.7 (2.3)	-1.3 (1.8)	-0.1 (0.1)
Inactives					
DS04644	C84640464	3.4 (1.8)	-2.8 (0.6)	-7.4 (1.0)	-14.7 (7.4)
DS05168	C04940516	11.1 (1.8)	-1 (1.6)	2.8 (1.6)	-12.4 (10.9)
DS18339	C71281833	15.3 (7.7)	-3.7 (0.3)	0.7 (1.1)	-21.8 (9.5)
DS34376	C95373437	16.1 (7.5)	-2 (0.8)	-1.6 (1.9)	-11.3 (4.2)
DS44245	C47934424	2.4 (14.7)	1.8 (4.4)	-3.4 (1.0)	-13.7 (10.5)
DS44738	C48954473	4.4 (14.0)	0.4 (1.7)	10.5 (23.6)	-20.8 (0.4)
DS57124	C17055712	23.9 (7.5)	7.8 (5.0)	-3 (1.8)	-20.2 (19.8)
DS59212	C09205921	7.8 (3.3)	0.2 (3.6)	5.8 (0.2)	-22.9 (4.9)
DS72975	C06807297	19.8 (5.0)	-1.1 (2.1)	9.8 (21.9)	-12.4 (1.8)
DS74417	C95387441	18 (3.2)	2.1 (0.4)	-2.8 (0.7)	-0.3 (1.6)
DS75739	C48697573	4.3 (6.9)	1.7 (0.8)	-0.6 (1.1)	-11.1 (4.9)
DS76514	C00137651	3.2 (10.7)	17.4 (0.4)	-0.6 (1.0)	-33.6 (18.1)
DS84326	C20858432	23.4 (0.6)	0.7 (0.8)	0.2 (2.8)	-8.2 (13.6)
DS99367	C71899936	17.3 (0.1)	1.4 (0.1)	-3.3 (0.5)	-19.6 (11.8)
DS23815	C71422381	10.7 (0.4)	-4,7 (3.1)[a]	-7.2 (5.7)	-19.7 (2.4)
DS31939	C02343193	9.6 (0.2)	-9,8 (7.8)[a]	-1.7 (0.8)	-27.8 (10.8)
DS52225	C44425222	22.7 (15.5)	0.9 (3.6)	4.5 (4.9)	-17.7 (15.6)
DS62156	C08706215	16.6 (0.4)	1.5 (5.8)	3.1 (3.3)	-6.3 (1.6)
DS74631	C07397463	11.3 (4.5)	20 (5.2)	-4.6 (2.9)	-22.8 (15.9)
DS82066	C02228206	12.6 (1.9)	3.6 (2.3)	-1.4 (0.8)	-18.5 (1.8)
DS11689	C02341168	18.1 (7.9)	-0.2 (0.4)	0.1 (2.1)	-8.3 (20.8)
DS66846	C02226684	14.5 (8.6)	4.6 (7.7)	-2.5 (1.6)	-18.7 (11.9)
DS74871	C07397487	23.3 (4.2)	22.1 (2.3)	-8.3 (1.2)	-44.9 (24.1)

[a] Compound interfered with assay readout.

3.2. Raw data for LigProfSim, PocSeqSim, IFPSim, and PocStructSim

3.2.1. LigProfSim

Table S4. LigProfSim matrix: Similarity values per kinase pair

kinase	EGFR	ErbB2	BRAF	CDK2	LCK	MET	p38a	KDR	p110a
EGFR	0.59	0.55	0.53	0.19	0.29	0.23	0.48	0.35	0.07
ErbB2	0.55	0.62	0.50	0.31	0.21	0.24	0.44	0.41	0.00
BRAF	0.53	0.50	0.82	0.36	0.58	0.39	0.74	0.77	0.33
CDK2	0.19	0.31	0.36	0.55	0.14	0.21	0.25	0.63	0.33
LCK	0.29	0.21	0.58	0.14	0.63	0.27	0.54	0.44	0.00
MET	0.23	0.24	0.39	0.21	0.27	0.79	0.11	0.55	0.00
p38a	0.48	0.44	0.74	0.25	0.54	0.11	0.77	0.53	0.00
KDR	0.35	0.41	0.77	0.63	0.44	0.55	0.53	0.70	0.18
p110a	0.07	0.00	0.33	0.33	0.00	0.00	0.00	0.18	0.65

Table S5. LigProfSim counts: Number of ChEMBL compounds commonly tested in each kinase pair

kinase	EGFR	ErbB2	BRAF	CDK2	LCK	MET	p38a	KDR	p110a
EGFR	5702	1199	70	47	129	82	46	875	180
ErbB2	1199	1690	22	29	28	29	9	189	1
BRAF	70	22	3625	14	38	31	42	268	3
CDK2	47	29	14	1520	22	24	8	122	12
LCK	129	28	38	22	1552	66	136	419	5
MET	82	29	31	24	66	2851	18	348	2
p38a	46	9	42	8	136	18	3581	125	5
KDR	875	189	268	122	419	348	125	7426	175
p110a	180	1	3	12	5	2	5	175	4150

Table S6. LigProfSim common actives: Number of ChEMBL compounds commonly active in each kinase pair

kinase	EGFR	ErbB2	BRAF	CDK2	LCK	MET	p38a	KDR	p110a
EGFR	3382	658	37	9	38	19	22	303	13
ErbB2	658	1048	11	9	6	7	4	77	0
BRAF	37	11	2968	5	22	12	31	207	1
CDK2	9	9	5	837	3	5	2	77	4
LCK	38	6	22	3	976	18	73	183	0
MET	19	7	12	5	18	2248	2	193	0
p38a	22	4	31	2	73	2	2753	66	0
KDR	303	77	207	77	183	193	66	5197	32
p110a	13	0	1	4	0	0	0	32	2706

3.2.2. PocSeqSim

Table S7. Kinase sequence identity of binding site residues (MSA of 85 binding site residues from KLIFS)

kinase	EGFR	ErbB2	BRAF	CDK2	LCK	MET	p38	PI3K	VEGFR2
EGFR	1	0.89	0.38	0.32	0.45	0.46	0.39	0.12	0.47
ErbB2	0.89	1	0.4	0.33	0.42	0.47	0.4	0.12	0.44
BRAF	0.38	0.4	1	0.33	0.39	0.38	0.38	0.16	0.4
CDK2	0.32	0.33	0.33	1	0.38	0.36	0.47	0.11	0.34
LCK	0.45	0.42	0.39	0.38	1	0.4	0.39	0.15	0.44
MET	0.46	0.47	0.38	0.36	0.4	1	0.36	0.12	0.47
p38	0.39	0.4	0.38	0.47	0.39	0.36	1	0.14	0.39
PI3K	0.12	0.12	0.16	0.11	0.15	0.12	0.14	1	0.15
VEGFR2	0.47	0.44	0.4	0.34	0.44	0.47	0.39	0.15	1

3.2.3. IFPSim

Table S8. IFPSim matrix: Similarity values per kinase pair

kinase1	EGFR	ErbB2	BRAF	CDK2	LCK	MET	p38a	PI3K	VEGFR2
EGFR	1.0	0.78	0.76	0.89	0.83	0.77	0.8	0.65	0.83
ErbB2	0.78	0.71	0.65	0.61	0.59	0.56	0.74	0.5	0.61
BRAF	0.76	0.65	0.96	0.79	0.97	0.87	0.96	0.52	0.93
CDK2	0.89	0.61	0.79	1.0	0.81	0.85	0.8	0.65	0.79
LCK	0.83	0.59	0.97	0.81	0.91	0.82	0.87	0.62	0.94
MET	0.77	0.56	0.87	0.85	0.82	1.0	0.76	0.57	0.87
p38a	0.8	0.74	0.96	0.8	0.87	0.76	1.0	0.53	0.96
PI3K	0.65	0.5	0.52	0.65	0.62	0.57	0.53	0.91	0.52
VEGFR2	0.83	0.61	0.93	0.79	0.94	0.87	0.96	0.52	1.0

3.2.4. PocStructSim

Table S9. PocStructSim matrix: Similarity values per kinase pair

	EGFR	ErbB2	VEGFR2	PI3K	BRAF	CDK2	LCK	MET	p38a
EGFR	1.000	0.400	0.478	0.241	0.518	0.500	0.534	0.451	0.466
ErbB2	0.400	1.000	0.291	0.164	0.318	0.300	0.373	0.279	0.259
VEGFR2	0.478	0.291	1.000	0.290	0.607	0.427	0.615	0.435	0.565
PI3K	0.241	0.164	0.290	1.000	0.259	0.282	0.225	0.242	0.322
BRAF	0.518	0.318	0.607	0.259	1.000	0.589	0.491	0.409	0.522
CDK2	0.500	0.300	0.427	0.282	0.589	1.000	0.456	0.460	0.433
LCK	0.534	0.373	0.615	0.225	0.491	0.456	1.000	0.433	0.419
MET	0.451	0.279	0.435	0.242	0.409	0.460	0.433	1.000	0.409
p38a	0.466	0.259	0.565	0.322	0.522	0.433	0.419	0.409	1.000
